# Hepatitis A outbreak disproportionately affecting men who have sex with men (MSM) in the European Union and European Economic Area, June 2016 to May 2017

**DOI:** 10.2807/1560-7917.ES.2018.23.33.1700641

**Published:** 2018-08-16

**Authors:** Patricia Ndumbi, Gudrun S Freidl, Christopher J Williams, Otilia Mårdh, Carmen Varela, Ana Avellón, Ingrid Friesema, Harry Vennema, Kazim Beebeejaun, Siew Lin Ngui, Michael Edelstein, Alison Smith-Palmer, Niamh Murphy, Jonathan Dean, Mirko Faber, Jürgen Wenzel, Mia Kontio, Luise Müller, Sofie Elisabeth Midgley, Lena Sundqvist, Josefine Lundberg Ederth, Anne-Marie Roque-Afonso, Elisabeth Couturier, Sofieke Klamer, Javiera Rebolledo, Vanessa Suin, Stephan W. Aberle, Daniela Schmid, Rita De Sousa, Gonçalo Figueiredo Augusto, Valeria Alfonsi, Martina Del Manso, Anna Rita Ciccaglione, Kassiani Mellou, Christos Hadjichristodoulou, Alastair Donachie, Maria-Louise Borg, Maja Sočan, Mario Poljak, Ettore Severi, Members of the European Hepatitis A Outbreak Investigation Team

**Affiliations:** 1 European Programme for Intervention Epidemiology Training (EPIET)| European Centre for Disease Prevention and Control, Solna, Sweden; 2 Public Health Wales, Cardiff, United Kingdom; 3 European Centre for Disease Prevention and Control, Solna, Sweden; 4 Instituto de Salud Carlos III, CIBER Epidemiología y Salud Pública, Madrid, Spain; 5 National Institute for Public Health and the Environment (RIVM), Bilthoven, the Netherlands; 6 Public Health England Colindale, London, United Kingdom.; 7 Health Protection Scotland, Glasgow, Scotland, United Kingdom; 8 Health Protection Surveillance Centre, Dublin, Ireland; 9 National Virus Reference Laboratory, Dublin, Ireland; 10 Robert Koch Institute, Berlin, Germany; 11 University Hospital Regensburg, Regensburg, Germany; 12 National Institute for Health and Welfare (THL), Helsinki, Finland; 13 Statens Serum Institut, Copenhagen, Denmark; 14 The Public Health Agency of Sweden, Stockholm, Sweden; 15 Centre National de Référence Virus des hépatites à transmission entérique, Villejuif, France; 16 Sante publique France, Saint-Maurice, France; 17 Sciensano, Brussels, Belgium; 18 Medical University of Vienna, Vienna, Austria; 19 Austrian Agency of Health and Food Safety, Vienna, Austria; 20 National Institute of Health Dr Ricardo Jorge, Lisbon, Portugal; 21 Directorate-General of Health, Lisbon, Portugal; 22 Instituto Superiore Di Sanita, Rome, Italy; 23 Hellenic Centre for Disease Control, Athens, Greece; 24 University of Thessaly, Larissa, Greece; 25 Health Promotion and Disease Prevention Directorate, Msida, Malta; 26 National Institute of Public Health, Ljubljana, Slovenia; 27 University of Ljubljana, Ljubljana, Slovenia; 28 The Members of the European Hepatitis A Outbreak Investigation Team have been listed at the end of this article

**Keywords:** hepatitis A, hepatitis A virus, men who have sex with men - MSM, vaccine-preventable diseases, vaccines and immunisation, sexually transmitted infections

## Abstract

Between 1 June 2016 and 31 May 2017, 17 European Union (EU) and European Economic Area countries reported 4,096 cases associated with a multi-country hepatitis A (HA) outbreak. Molecular analysis identified three co-circulating hepatitis A virus (HAV) strains of genotype IA: VRD_521_2016, V16–25801 and RIVM-HAV16–090. We categorised cases as confirmed, probable or possible, according to the EU outbreak case definitions. Confirmed cases were infected with one of the three outbreak strains. We investigated case characteristics and strain-specific risk factors for transmission. A total of 1,400 (34%) cases were confirmed; VRD_521_2016 and RIVM-HAV16–090 accounted for 92% of these. Among confirmed cases with available epidemiological data, 92% (361/393) were unvaccinated, 43% (83/195) travelled to Spain during the incubation period and 84% (565/676) identified as men who have sex with men (MSM). Results depict an HA outbreak of multiple HAV strains, within a cross-European population, that was particularly driven by transmission between non-immune MSM engaging in high-risk sexual behaviour. The most effective preventive measure to curb this outbreak is HAV vaccination of MSM, supplemented by primary prevention campaigns that target the MSM population and promote protective sexual behaviour.

## Background

Hepatitis A (HA) is an acute liver disease caused by the hepatitis A virus (HAV) [1]. Transmission is faecal-oral via consumption of contaminated food or water or through direct person-to-person contact, including sexual contact (particularly oro-anal, digito-anal and genito-oral sex). The mean incubation period is 28 days (range: 15–50). Laboratory diagnosis is based on the detection of serological (anti-HAV IgM) or molecular (HAV RNA) markers of acute HAV infection. Preschool children are usually asymptomatic; however, most adults experience symptoms such as fever, diarrhoea and acute jaundice [2]. Fulminant hepatic failure and death are rare (0.3% of clinical cases) [3].

Men who have sex with men (MSM) are particularly at risk for HAV infection [4]. Over the past two decades, HA outbreaks among MSM have been reported across Australia, Europe and North-America [5,6]. The last reported European HA outbreak among MSM in 2008–09 involved a high proportion of HIV-positive MSM [4,6-8]. HAV and HIV infections among MSM share overlapping risk factors such as casual sex with multiple partners, which often involves injecting drug use and unprotected sexual intercourse [9]. Furthermore, HIV co-infection can exacerbate HAV-associated liver abnormalities and prolong the faecal excretion of HAV [10].

A safe and effective vaccine against HAV has been available since 1995 [11]. In low and very low endemicity settings such as Europe, the World Health Organization (WHO) recommends HAV vaccination for vulnerable populations such as MSM; however, vaccine coverage is not known [12].

## Outbreak detection

Between October 2016 and January 2017, the United Kingdom (UK), the Netherlands and Germany signalled an increase in HA cases among MSM, via the European Union (EU) Early Warning and Response System (EWRS) and the Epidemic Intelligence Information System for Food and Waterborne Diseases and Zoonoses (EPIS-FWD) of the European Centre for Disease Prevention and Control (ECDC), both of which facilitate information sharing on outbreaks and potential health threats. Molecular investigations detected three distinct HAV genotype IA strains: VRD_521_2016, RIVM-HAV16–090 and V16–25801. Following these alerts, 14 other EU/EEA countries notified ECDC of HA cases among MSM. ECDC convened a multistate outbreak investigation team in December 2016.

We report on this Europe-wide investigation of a multi-strain HA outbreak to describe its extent and its characteristics within affected EU/EEA countries and to identify strain-specific risk factors associated with transmission.

## Methods

### Study design

All European countries affected by this outbreak were invited to contribute to the multistate outbreak investigation consisting of a descriptive study and a case–case study.

#### Descriptive study

We conducted a retrospective descriptive study with the aim to describe characteristics of the population affected based on country-specific HA attack rates (AR) and male-to-female (M:F) ratios for a fixed period in each year, from 2012 to 2017. We used M:F ratios as indicators of MSM-associated epidemics where data on sexual orientation was not available [13]. MSM-status was defined as self-identifying as MSM or reporting sexual contact with another man. We also described the characteristics of confirmed, probable and possible outbreak cases notified between 1 June 2016 and 31 May 2017. Results are presented as percentages of cases with available information.

#### Case–case study

The case–case study aimed to test the hypothesis that cases infected with any of the outbreak strains differed in exposures related to sexual transmission, including sexual practices, travel history and use of dating apps.

### European outbreak case definition

Outbreak cases were laboratory-confirmed HAV infections in EU/EEA residents, with a date of symptom onset (or sample date, where missing) between 1 June 2016 and 31 May 2017. The exposure period for defining probable and possible cases was 8 weeks before symptom onset or sample date.

Confirmed cases were those with a minimum sequence length of 300 nucleotides (nt) that was at least 99.3% homologous to one of the three outbreak strains based on overlapping fragments at the VP1–2A region. Cases epidemiologically linked to non-outbreak strain cases (< 99.3% homology) were excluded. The case definition was applied at country level and the number of excluded cases has not been collected.

Case definitions for probable and possible cases are included in Supplement 1.

### Data collection

HA is a notifiable disease in all participating EU/EEA countries. Local public health departments investigated HA cases for demographic, clinical and exposure characteristics, then reported to national surveillance systems. For all countries data from mandatory notifications was used and assumed to be comprehensive at the national level. Reporting practices were similar across countries and had not changed since 2015.

For the descriptive study, we asked national focal points for hepatitis A to provide aggregated numbers of all HA cases, by sex and age group, reported from 1 June to 31 March between 2012 and 2017 (used for AR and M:F ratio calculation). National focal points were also asked to complete a line list of all confirmed, probable and possible cases; information on criteria for meeting case definition, onset date, sex, age, travel history, hospitalisation, vaccination, HIV status and MSM status was also requested. In addition, we collected information on national HAV vaccination guidelines targeted to MSM and HIV-positive individuals.

For the case–case study, we compared the three outbreak strains for cases reported from July 2016 to May 2017. We developed a standardised European questionnaire, adapted from the Public Health England (PHE) enhanced surveillance form [14] to collect information on the number of sexual partners, visiting and/or having sex in gay venues and travel history during the 8 weeks preceding symptoms onset. Information on condom use, HIV co-infection and use of HIV-pre-exposure prophylaxis (PrEP) was also collected. Countries were asked to complete the questionnaire for confirmed outbreak cases who agreed to provide information. The European questionnaire was implemented in Belgium, Denmark, Greece, Italy and the UK (with the exception of England). England used the earlier PHE version and the Netherlands used a similar questionnaire, also adapted from the PHE questionnaire.

### Virological analysis

HAV was confirmed through detection of anti-HAV IgM or by PCR identification of HAV RNA in local laboratories, followed by sequencing in national reference laboratories. The sampling strategy for sequencing differed across countries; most participating countries sequenced a subset of samples from notified cases, with only a few countries routinely sequencing all samples from confirmed notified cases (Denmark, Finland, Ireland, the Netherlands and the UK). Norway sequenced all outbreak-associated samples; Malta did not have sequencing capacity.

Sequencing of the VP1–2a region was performed according to national protocols, and followed by comparison to the reference sequences of each of the three outbreak strains [15-17]; 93% of sequences were ≥ 400 nt.

### Statistical analysis

We calculated country-specific attack rates per 100,000 residents. Population data were extracted from Eurostat [18]. We also calculated the M:F ratio for HA cases aged 18–45 years.

Categorical variables were compared using the chi-squared test. Continuous variables were expressed as median with the corresponding interquartile (IQR) range and were compared using the Kruskal-Wallis test. P values of < 0.05 were considered statistically significant.

We developed four logistic regression models to detect associations between exposure variables and specific outbreak strains. Model-1 compared VRD_521_2016 to RIVM-HAV16–090, model-2 compared VRD_521_2016 to V16–25801, model-3 compared RIVM-HAV16–090 to V16–25801 and model-4 compared the RIVM-HAV16–090/V16–25801 combination (grouped due to having similar geographical distributions) to VRD_521_2016. All models included age, vaccination status, number of sexual partners, travel abroad and use of dating apps. Since all four regression models provided similar results, we only reported odds ratios (OR) and 95% confidence interval (CI) obtained from model-4.

Case mapping by country of reporting was done using ArcGIS Desktop software.

## Results

Seventeen countries contributed data for the outbreak investigation: Austria, Belgium, Denmark, Finland, France, Germany, Greece, Ireland, Italy, Malta, the Netherlands, Norway, Portugal, Slovenia, Spain, Sweden and the UK (England, Wales, Northern Ireland and Scotland). A total of 4,096 outbreak cases were reported (1,400 confirmed, 964 probable and 1,732 possible).

### Descriptive study

#### Aggregated data

All countries except Norway provided aggregated data. Between 1 June 2016 and 31 March 2017, the national incidence of HA more than doubled in Austria, Greece, Italy, Malta, Portugal, Spain and the UK, compared with the same period during the previous 4 years. For 11 of the 16 countries, the M:F ratio was at least 3.0 (range: 0.7–9.5) during the outbreak period. Spain was the most affected country with an attack rate of 4.4 cases per 100,000 population (a fourfold increase from the previous 4 years) and a M:F ratio of 7.5 (Table 1).

**Table 1:** Total number, attack rate and male to female ratio of hepatitis A patients notified between 1 June and 31 March, 2012–2017, participating European Union/European Economic Area countries

Country^a, b^	Number of hepatitis A cases (all ages)		Attack rate (all ages)^c^		Male:Female ratio (18–45 years)^d^	
	2012–16^e^ (Average)	2016–17^e^	2012–16^e^ (Average)	2016–17^e^	2012–16^e^ (Average)	2016–17^e^
Austria	60	125	0.7	**1.4**	1.1	**4.6**
Belgium	148	223	1.3	2.0	1.2	**4.7**
Denmark	42	29	0.7	0.5	1.6	1.3
Finland	23	17	0.4	0.3	1.1	**6.0**
France	787	750	1.2	1.1	1.1	**3.1**
Germany	720	668	0.9	0.8	1.3	**3.3**
Greece	84	241	0.8	**2.2**	1.1	**3.3**
Italy	421	976	0.7	**1.6**	1.5	**8.4**
Ireland	22	41	0.5	0.9	1.3	0.7
Malta	2	6	0.5	1.4	1.0	1.5
The Netherlands	96	119	0.6	0.7	1.1	**3.0**
Portugal	19	164	0.2	**1.6**	1.6	**9.5**
Slovenia	8	11	0.4	0.5	1.7	2.0
Spain	531	2,039	1.1	**4.4**	1.2	**7.5**
Sweden	85	84	0.9	0.9	1.0	1.2
UK	265	553	0.4	**0.9**	1.0	**4.7**

MSM: men who have sex with men; UK: United Kingdom.

^a^ A food-borne HA outbreak occurred in Denmark, Finland, Norway and Sweden in 2012/13.

^b^ A food-borne HA outbreak occurred in Austria, England, Finland, France, Germany, Ireland, Italy, the Netherlands, Norway and Sweden in 2013/14.

^c^ The 2016/17 attack rates with ≥ 2 fold increase from previous 4 years are in bold.

^d^ The 2016/17 M:F ratios ≥ 3 are in bold.

^e^ For all years, the period referred to is 1 June to 31 March (of the subsequent year).

### Confirmed cases

In total, 1,400 confirmed cases were reported across all countries except Malta. The number of confirmed cases per country ranged between 1 and 294. The completeness of variables varied considerably; 1,382/1,400 cases (99%) had information on sex, compared with 140/1,400 (10%) for HIV status.

Ninety-three percent (n = 1,283/1,382) were male, 79% (n = 1,086/1,378) were aged between 18 and 45 years and 54% (n = 429/797) were hospitalised. Forty-three percent (n = 60/140) with responses or 4.3% of all confirmed cases were reported as HIV-positive (Table 2).

**Table 2:** Characteristics of confirmed hepatitis A cases by strain in multi-strain outbreak affecting predominantly MSM, 1 June 2016–31 May 2017, participating European Union/European Economic Area countries (n = 1,400 cases)

Characteristics	RIVM-HAV16–090		V16–25801		VRD 521_2016		Total	
	n	% ^a^	n	% ^a^	n	% ^a^	n	% ^a^
** Total**	**495**	**35**	**119**	**9**	**786**	**56**	**1,400**	**100**
**Sex**								
Male	448	92	112	94	723	93	1,283	93
**Age**								
Median, IQR	34	28–45	34	28–40	33	28–42	33	28–43
**Age categories^b^**								
0–17 years	14	3	2	2	10	1	26	2
18–45 years	358	73	96	83	632	82	1,086	79
46–65 years	101	21	18	16	125	16	244	18
66+ years	15	3	0	0	7	1	22	2
**Subtotal**	**488**	**100**	**116**	**100**	**774**	**100**	**1,378**	**100**
**MSM**								
Yes	239	81	41	87	284	86	564	84
**HIV infection**								
Yes	28	42	1	20	31	45	60	43
**Hospitalisation**								
Yes	195	57	39	46	195	52	429	54
**Reporting country^b^**								
Austria	24	92	1	4	1	4	26	100
Belgium	31	74	1	2	10	24	42	100
Denmark	0	0	1	33	2	67	3	100
Finland	1	13	1	13	6	75	8	100
France	113	38	8	3	173	59	294	100
Germany	26	27	41	42	31	32	98	100
Greece	0	0	2	50	2	50	4	100
Ireland	1	14	0	0	6	86	7	100
Italy	45	26	5	3	125	71	175	100
The Netherlands	46	49	8	9	39	42	93	100
Norway	0	0	0	0	1	100	1	100
Portugal	1	1	0	0	108	99	109	100
Slovenia	3	75	0	0	1	25	4	100
Spain	27	10	12	5	223	85	262	100
Sweden	9	82	0	0	2	18	11	100
UK	168	65	39	15	56	21	263	100
**Subtotal**	**495**	** NA**	**119**	** NA**	**786**	**NA**	**1,400**	**NA**
**Travel history^b^**								
Yes	100	31	24	30	71	19	195	25
**Country of travel**								
Spain	35	35	11	46	37	52	83	43
Germany	11	11	2	8	3	4	16	8
Belgium	6	6	0	0	2	3	8	4
Portugal	3	3	1	4	4	6	8	4
Italy	1	1	1	4	5	7	7	4
Other: EU	14	14	3	13	8	11	25	13
Other: non-EU	22	22	3	13	9	13	34	17
Multiple EU	8	8	5	21	7	10	20	10
Multiple EU/non-EU	4	4	0	0	4	6	8	4
**Subtotal**	**104**	**NA**	**26**	** NA**	**79**	** NA**	**209**	**NA**

EU: European Union; IQR: interquartile range; MSM: men who have sex with men; NA: not applicable; UK: United Kingdom.

^a^ All percentages are based on the total of available data for each variable.

^b^ P values < 0.05.

P values are based on comparison between the three HAV strains using the chi-squared test for categorical variables and the Kruskal-Wallis test for continuous variables.

The proportion of hospitalisations did not significantly differ between HIV-positive and HIV-negative cases (45% vs 55%, p = 0.89). Among male cases, 84% (n = 565/676) were MSM and 49% (n = 50/102) of MSM with known HIV status were HIV-positive. Non-MSM cases (n = 159) did not significantly differ from MSM cases regarding travel history (p = 0.47).

Ninety-two percent (n = 361/393) of cases were unvaccinated. Of the remaining cases (n = 32), eight had received only one dose of hepatitis A vaccine (three cases received it 2 weeks before symptoms onset), three cases had received two doses (two were HIV-positive patients) and the remaining 21 lacked information on the number of doses received. A quarter of confirmed cases (n = 195/782) travelled outside the reporting country during the incubation period, with Spain being the most visited country (n = 83; 43%).

**Figure 1:**
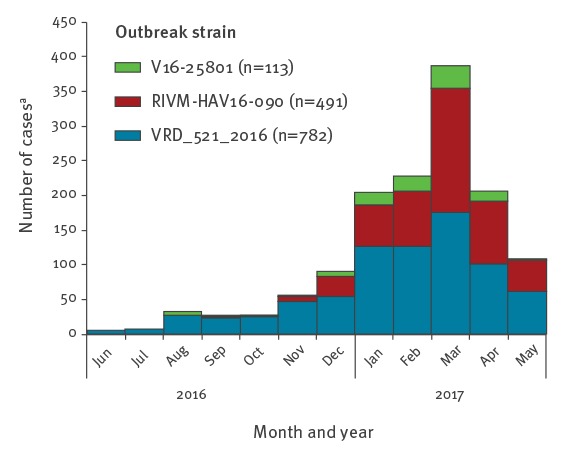
Confirmed hepatitis A cases by strain and date of reporting in multi-strain outbreak affecting predominantly MSM, 1 June 2016–31 May 2017, participating European Union/European Economic Area countries, (n = 1,386 cases)

^a^ There are 14 cases not displayed because the exact onset date could not be identified.

**Figure 2:**
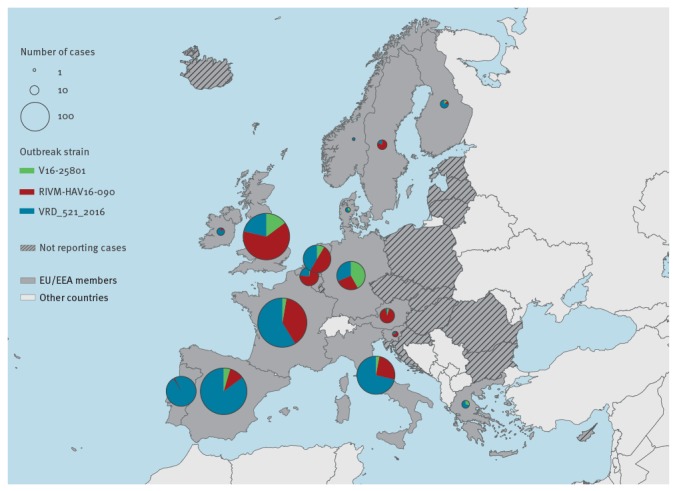
Confirmed hepatitis A cases by strain and geographical distribution in multi-strain outbreak affecting predominantly MSM, 1 June 2016–31 May 2017, participating European Union/European Economic Area countries (n = 1,400 cases)

The epidemic curve indicates a sustained increase in confirmed case numbers until March 2017 (Figure 1). The most frequently reported strain was VRD_521_2016 (n = 786 /1,400; 56%), which was the first outbreak strain detected. Strain distribution varied geographically. In southwestern Europe the predominant strain was VRD_521_2016 (n = 458/550; 83%). RIVM-HAV16–090 was the main strain in central Europe and the UK (n = 396/752; 53%), except in Germany where V16–25801 was the most prevalent (n = 41/98; 42%) (Figure 2).

Map produced on 17 January 2018. Administrative boundaries: ^©^EuroGraphics, ^©^UN-FAO.

Case characteristics did not differ between strains with regards to median age (p = 0.24), MSM status (p = 0.21), HIV status (p = 0.551) and hospitalisation (p = 0.13). VRD_521_2016 cases, which account for 85% of the outbreak strains reported from Spain, were less likely to have travelled during the incubation period (p = 0.001).

### Confirmed, probable and possible outbreak cases

Most of the 4,096 outbreak cases (including probable and possible cases) were reported in Spain (n = 2,128; 52%), Italy (n = 797; 19%) and France (n = 294; 7%). Denmark, Ireland, Finland, Malta, Norway, Slovenia and Sweden reported fewer than 15 cases each (Table 3). Cases clustered in major cities like London, Amsterdam, Berlin, Lisbon and Vienna; countries with the earliest onset dates were Spain, Italy and the UK.

**Table 3:** Characteristics of hepatitis A cases by case classification in multi-strain outbreak affecting predominantly MSM, 1 June 2016–31 May 2017, participating European Union/European Economic Area countries (n = 4,096)

Characteristics	Confirmed		Probable		Possible		Total	
	n	% ^a^	n	% ^a^	n	% ^a^	n	% ^a^
** Total**	**1,400**	**34**	**964**	**24**	**1,732**	**42**	**4,096**	**100**
**Sex**								
Male	1,283	93	964	100^b^	1,732	100^b^	3,979	98
**Age**								
Median (IQR)	33	28–43	33	27–40	32	26–38	32	27–40
**Age categories**								
0–17 years	26	2	4	0	NA	NA	30	1
18–45 years	1,086	79	857	89	1,732	100^b^	3,675	90
46–65 years	244	18	106	11	NA	NA	350	8
66+ years	22	1	1	0	NA	NA	23	1
**Subtotal**	**1,378**	** 100**	**963**	**100**	**1,732**	**100**	**4,073**	**100**
**Reporting country**								
Austria	26	2	NA	NA	53	3	79	2
Belgium	42	3	36	4	40	2	118	3
Denmark	3	0	4	0	NA	NA	7	0
Finland	8	1	NA	NA	NA	NA	8	0
France	294	21	NA	NA	NA	NA	294	7
Germany	98	7	NA	NA	NA	NA	98	2
Greece	4	0	23	2	15	1	42	1
Ireland	7	1	5	1	NA	NA	12	0
Italy	175	13	343	36	279	16	797	19
Malta	NA	NA	7	1	2	0	9	0
The Netherlands	93	7	10	1	13	1	116	3
Norway	1	0	NA	NA	NA	NA	1	0
Portugal	109	8	NA	NA	NA	NA	109	3
Slovenia	4	0	NA	NA	NA	NA	4	0
Spain	262	19	536	56	1,330	77	2,128	52
Sweden	11	1	NA	NA	NA	NA	11	0
UK	263	19	NA	NA	NA	NA	263	6
**Subtotal**	**1,400**	**100**	**964**	**100**	**1,732**	**100**	**4,096**	**100**

NA: not applicable.

^a^ All percentages are based on the total of available data for each variable.

^b^ As per outbreak case definition.

### Case–case study

Enhanced surveillance information was reported for 308 cases from seven countries, the UK accounting for 70% of these (Table 4). Among cases with available information on the number of sexual partners, 72 (36%) reported sexual contacts with at least three partners and 39 were with anonymous partners. In the 8 weeks preceding illness, 51 of 133 MSM reported consistent condom use during sex. Of those reporting attending gay clubs or saunas, 13/60 and 32/35 reported sexual contact in these venues, respectively.

**Table 4:** Characteristics and exposures of hepatitis A cases during the 8 weeks preceding symptoms onset in multi-strain outbreak affecting predominantly MSM, 1 June 2016–31 May 2017, participating European Union/European Economic Area countries (n = 308)

Characteristics	RIVM-HAV16-090		V16-25801		VRD 521_2016		Not-sequenced		Total	
	n	% ^a^	n	% ^a^	n	% ^a^	n	% ^a^	n	% ^a^
**Total**	**166**	**54**	**39**	**13**	**86**	**28**	**17**	**5**	**308**	**100**
**Sex**										
Male	155	95	38	97	79	92	17	100	289	94
**Age categories**										
0–17 years	7	4	0	0	0	0	1	6	8	3
18–45 years	137	83	36	92	66	77	15	88	254	82
46–65 years	21	13	3	8	18	21	1	6	43	14
66 + years	1	1	0	0	2	2	0	0	3	1
**Subtotal**	**166**	**100**	**39**	**100**	**86**	**100**	**17**	**100**	**308**	**100**
**Sexual contact^b^**										
With male	119	96	27	90	49	75	27	90	212	90
With female	4	3	3	10	6	9	3	10	13	6
With both	0	0	0	0	3	5	0	0	3	1
No sexual exposure	1	1	0	0	7	11	0	0	8	3
**Subtotal**	**124**	**100**	**30**	**100**	**65**	**100**	**30**	**100**	**236**	**100**
**No of sexual contacts**										
1–2	77	70	14	50	32	63	4	40	127	64
≥ 3	33	30	14	50	19	37	6	60	72	36
**Subtotal**	**110**	**100**	**28**	**100**	**51**	**100**	**10**	**100**	**199**	**100**
**Regular sex partners^c^**										
1–2	50	96	13	81	22	100	4	100	89	95
3–5	2	4	2	13	0	0	0	0	4	4
6–10	0	0	1	6	0	0	0	0	1	1
> 10	0	0	0	0	0	0	0	0	0	0
**Subtotal**	**52**	**100**	**16**	**100**	**22**	**100**	**4**	**100**	**94**	** 100**
**Casual sex partners^c^**										
1–2	34	65	9	75	11	52	6	86	60	65
3–5	12	23	1	8	8	38	1	14	22	24
6–10	3	6	1	8	2	10	0	0	6	7
> 10	3	6	1	8	0	0	0	0	4	4
**Subtotal**	**52**	**100**	**12**	**100**	**21**	**100**	**7**	**100**	**92**	**100**
**Anonymous sex partners^c^**										
1–2	24	60	4	33	8	47	0	0	36	48
3–5	9	23	3	25	8	47	2	33	22	29
6–10	3	8	3	25	0	0	2	33	8	11
> 10	4	10	2	17	1	6	2	33	9	12
**Subtotal**	**40**	**100**	**12**	**100**	**17**	**100**	**6**	**100**	**75**	**100**
**Condom use**										
Always	28	36	8	36	13	54	2	20	51	38
Most time (> 50%)	12	16	0	0	1	4	6	60	19	14
Sometime (< 50%)	18	23	6	27	4	17	1	10	29	22
Never	19	25	8	36	6	25	1	10	34	26
**Subtotal**	**77**	**100**	**22**	**100**	**24**	**100**	**10**	**100**	**133**	**100**
**Use of dating apps to meet sex partners**										
Yes	35	31	5	24	13	26	5	50	58	30
**Attended a gay club**										
Yes	35	97	9	90	8	80	8	100	60	94
**Attended a gay sauna**										
Yes	24	100	3	75	3	75	5	100	35	95
**PrEP use**										
Always	0	0	0	0	0	0	1	11	1	0
Sometime (< 50%)	0	0	0	0	0	0	1	11	1	0
Never	130	100	26	100	64	100	7	78	227	99
**Subtotal**	130	**100**	26	**100**	64	**100**	9	**100**	229	**100**
**HIV infection**										
Yes	5	21	1	11	5	14	3	20	14	17
**Travel abroad**										
Yes	39	25	10	29	25	31	10	59	84	29
**Sex while abroad**										
Yes	16	64	4	80	11	58	8	89	39	67
**Reporting country**										
Belgium	0	0	0	0	0	0	10	59	10	3
Denmark	0	0	0	0	1	1	0	0	1	0
Greece	0	0	2	5	2	2	0	0	4	1
Italy	13	8	0	0	27	31	0	0	40	13
The Netherlands	13	8	2	5	7	8	7	41	29	9
Spain	0	0	5	13	3	3	0	0	8	3
UK	140	84	30	77	46	53	0	0	216	70
**Subtotal**	**166**	**100**	**39**	**100**	**86**	**100**	**17**	**100**	**308**	**100**

PrEP: pre-exposure prophylaxis; UK: United Kingdom.

^a^ Percentages are based on the total of available data for each variable.

^b^ P values < 0.05.

^c^ There is a slight difference in totals for type of sex partners (regular, casual and anonymous) as the first question on how many sex partners the patient has had is independent from the other questions on the number by type of sex partners.

P-values are based on comparison between the three HAV strains using the chi-squared test.

Of 308 cases, 84 reported travel abroad, of which 26 visited Spain. Information on sexual activity was available for 58 cases, of which 39 reported sex while abroad. Of 12 cases with available information, 10 reported attending Lesbian, Gay, Bisexual, Trans and Queer (LGBTQ) festivals. Of those with information on hospitalisation (n = 69), 66% percent (n = 177/269) were hospitalised for a median of five (IQR: 2–7) nights. Of 100 cases, 30% (n = 30) worked in the food or healthcare sector.

Of the 308 cases, 291 had sequence information (confirmed cases): 57% (n = 166/291) were RIVM-HAV16–090, 30% (n = 86/291) VRD_521_2016 and 13% (n = 39/291) V16–25801. Of these 291 cases, 44% (n = 129/291) had complete information on key exposures and were included in the regression analysis. Taking age and vaccination status into account, RIVM-HAV16–090 or V16–25801 cases did not statistically differ from VRD_521_2016 cases (reference) in terms of having ≥ 3 sexual partners (OR: 0.56; 95% CI: 0.23–1.36), travelling abroad (OR: 0.69; 95% CI: 0.28–1.69) and using dating apps for sexual encounters (OR: 1.55; 95% CI: 0.62–3.89) during the incubation period.

### National vaccination guidelines

Fourteen countries (Belgium, Denmark, Finland, France, Germany, Greece, Ireland, Italy, the Netherlands, Portugal, Slovenia, Spain, Sweden and the UK) provided data on their national vaccination guidelines, which differed across countries. All countries, except for Sweden, recommended HAV vaccination for MSM; in Finland, the Netherlands and Portugal, this recommendation only took effect during the outbreak described here. In the UK, the recommendation was restricted to times and places where transmission is high, but was extended during the current outbreak to all MSM attending sexual health services. Germany, Greece, Slovenia and the UK recommended HAV vaccination for HIV-positive individuals. Vaccination was free for MSM except in Belgium, Denmark, France and the Netherlands.

## Discussion

We document a large HA outbreak, with 1,400 confirmed cases infected with one of three identified outbreak strains and almost exclusively detected among young adult males. In total, there were 1,283 male cases with 676 (53%) providing information on sexual preference, of these 84% identified as MSM. Strain VRD_521_2016 was the first to be reported [15] and remains the most prevalent, particularly in southern Europe.

Strain evolution in HAV is very slow; the frequency of single nucleotide variants is generally low and proportional to the number of infections. In this outbreak, we observed three main variants of the outbreak strains: two with one nucleotide difference and one with two nucleotides difference (data not shown). This indicates repeated transmission during the outbreak, which is expected considering that it has been active for over a year. Double nucleotide variants were found mainly in the VRD_521_2016 strain.

As MSM were particularly affected by this outbreak, we estimated the likely scale of transmission among this group by defining probable and possible outbreak cases. Since June 2016 there have been over 4,000 cases reported in 17 EU/EEA countries linked to the current outbreak. Spain was the most affected country, accounting for over half of reported cases. Confirmed cases decreased after March 2017, but this was more likely attributable to a lag in the reporting of typing data than an indication of a change in the outbreak evolution.

The outbreak is ongoing as of March 2018 and, given that MSM appear to be disproportionately affected, it is possible that further transmission events may occur during summer Pride events across Europe in 2018 [19].

Although the strain distribution varied over time and country, we found no differences in exposures and demographic characteristics. It is thought that this outbreak could be driven by multiple introductions and prolonged transmission of HAV strains originating from different parts of the world, including Central America (VRD_521_2016) and Pacific-Asia (RIVM-HAV16-090). Reports of these strains in Canada (personal communication Meghan Hamel, Public Health Agency of Canada, July 2017), Israel, the US and Taiwan indicate that the outbreak also involves non-EU/EEA countries [16,20-22]. Between June 2016 and mid-May 2017, Chile also reported an increase of HA cases among MSM, but sequencing information was unavailable at the time of this study [20]. Between 1997 and 2005, closely related HAV strains circulated internationally, almost exclusively among MSM [23]. This suggests that without appropriate interventions large MSM networks can sustain transmission of HAV.

MSM are more likely to engage in high-risk sexual behaviour while travelling abroad [24]. Results from the European Men-Who-Have-Sex-With-Men Internet Survey (EMIS) conducted in 2010 show that 26% of European MSM reported sex abroad in the previous year, with Spain and Germany being the most common destinations [24]. Our results, 8 years later, show a higher proportion of MSM reporting sex abroad (n = 39/58, 67%). The increased incidence of HA within vulnerable and highly interconnected subgroups of MSM, along with the accumulation of susceptible individuals during the period between large outbreaks, are likely to fuel the rapid international spread of HAV through sexual transmission.

We found that a high proportion of cases engaged in unprotected sex with non-steady partners. Avoidance of faecal-oral exposure during sexual activity and safer sex practices (e.g. use of barrier methods) play an important part in the prevention of HAV and other sexually transmitted infections (STIs), including enteric STIs. It has been found previously that notifications of gonorrhoea, syphilis and shigellosis among MSM are increasing in Europe [25,26]. Similarities in age, HIV prevalence and geographical clustering between the HA cases reported in our study and men affected by these STIs could suggest that these infections primarily circulate within the same European MSM population. Information on STIs other than HIV was not assessed in this study; however, as HIV-status was poorly reported and numbers were small, caution is needed when interpreting the HIV prevalence.

As the outbreak continues to expand among MSM, there is an increased risk for spread to the wider population. During and after summer 2017, several countries reported small food-borne outbreaks associated with one of the outbreak strains, with limited increases of HA among females, children and the elderly reported [19].

Although HA is generally a self-limiting disease, it can cause both a substantial health and economic burden including hospitalisation, acute hepatic failure and work sickness absence. In this study, over half of the cases with available information were hospitalised for approximately a week. This is likely to be an overestimation, however, as there is a higher case ascertainment in hospitalised cases.

In the EU/EEA, decreased HAV circulation in the last decades has resulted in a large fraction of the population having no immunity triggered by natural infection, leaving them susceptible to contracting the infection [27]. HAV vaccination can compensate for this immunity gap by protecting susceptible individuals. Due to the extent of this outbreak, it could be hypothesised that HAV vaccine coverage among MSM is low despite being recommended by WHO and ECDC [12,27,28]; of the 17 affected countries in our study, only four recommended HAV vaccination for HIV-positive populations. Improving the uptake of HA vaccination within susceptible MSM and HIV-positive individuals could avoid protracted transmission within and from these groups [29]. However, during the outbreak, a global shortage of HA vaccine has affected several EU countries (Austria, Denmark, France, Italy, Portugal, Spain, Sweden and the UK) and this may have hampered vaccine delivery.

A limitation in this study is the potential underestimation of the extent of the outbreak, as only symptomatic individuals who sought care were reported. Confirmed cases were only those with the three strains originally reported, thus excluding other HAV strains potentially circulating among MSM. As of September 2017, the circulation of non-outbreak strains among MSM has only been reported in five cases (data not shown). Some variation of the outbreak strains may have been missed, given that sequence information was only limited to a small part of the genome. Moreover, sequencing capacity varied between countries, with Belgium, France, Italy, Portugal and Spain, only sequencing a proportion of cases (data not shown) and other European countries lacking sequencing capacity altogether. Since the definition of probable and possible cases was mostly applicable to adult males, cases in females or children may have been missed during the outbreak. The low completeness of some variables may have biased our estimates, particularly for MSM and HIV status, which are not routinely recorded in many countries. We also lacked comparative information from a control group.

The case–case design may have resulted in an underestimation of the odds ratios since cases used as controls may not accurately reflect the source population and the completeness of information related to the main variable of interest was suboptimal. Furthermore, a disproportionate number of cases with exposure information were from the UK and may not be generalisable to all outbreak cases. However, the similarities in exposures from the three strains support the common public health recommendations applied across affected countries.

The HA vaccine is a safe, effective and affordable preventive measure against HA in EU/EEA countries, and routine targeted HAV vaccination is generally recommended for MSM [27,28] (Supplement 2). However, a global vaccine shortage may have hampered the wide administration of the HA vaccine to MSM, which should be given priority when dealing with the vaccine shortage, especially during an outbreak. In order to mitigate the effect of the shortage, some authorities have also endorsed the off-label use of vaccination (e.g. administration of paediatric formulation vaccine to adults) [30].

Countries should also consider provision of post-exposure prophylaxis to identified sexual and other close contacts of HA cases, as well as delivery of messages to raise awareness among MSM on how unprotected sex increases the risk of STIs, including HA and HIV, and the importance of regular testing to prevent further transmission. The popularity of LGBTQ festivals provides excellent opportunities to deliver such messages. Given that many cases are at high risk for other STIs, testing for concurrent STIs (including HIV) should also be promoted.

Countries should also assess whether there is a need to enhance HA surveillance to ensure timely case detection and monitoring of this outbreak, such as its spread to other risk groups or the general population. So far, no outbreak-associated deaths have been reported, however the quality of outcome monitoring is likely sub-optimal.

## Conclusion

Our results suggest that a combination of international travel and sexual networks can sustain a large outbreak of HAV, with multiple virus strains, within a susceptible population. Our findings highlight the importance of recording sexual history and HIV status when investigating male HA patients.

The inclusion of molecular characterisation in the outbreak case definition enabled the linking of cases occurring at different times and geographical locations; thus demonstrating the added value of utilising molecular epidemiology. Since the joint investigation of the 2013–14 EU/EEA food-borne HA outbreak, the harmonisation of HAV sequencing according to the HAVnet protocol has facilitated data comparison among European laboratories. Typical measures for preventing most STIs (e.g. condom use or personal hygiene instructions) are not sufficient to prevent HA transmission altogether, but are nonetheless an important part of information campaigns for MSM with high-risk sexual behaviour. Furthermore, a safe and effective vaccine exists, and its use is recommended to prevent further transmission and future outbreak.

## Notes

**Members of the European Hepatitis A Outbreak Investigation Team:** Belgium: Sofie Parmentier, Valeska Laisnez, Alice Reynaerts, Romain Mahieu, Carole Schirvel; Denmark: Hanne Thang Vestergaard; France: Athinna Nisavanh; Germany: Heiko Jessen, Kai Michaelis, Dagmar Sissolak, Janine Thoulass, Ruth Zimmermann; Ireland: Lelia Thornton; Italy: Domenico Martinelli, Maria Giovanna Cappelli, Giulia Del Matto, Maria Chironna, Roberto Bruni; Malta: Maria-Louise Borg; Portugal: Isabel Aldir, Marina Nascimento, António Martins, Bárbara Aguiar, Diogo Medina; Slovenia: Mario Fafangel, Alenka Trop Skaza, Jana Svetičič Marinko, Dražen Stojanovič, Tanja Selič Kurinčič; Spain: Carmen Montaño-Remacha, Marcelino Garcia Fernandez, Diana Gomez-Barroso, Sonia Vazquez, Noelia Reyes; United Kingdom: Sema Mandal, Koye Balogun; European Centre for Disease Prevention and Control (ECDC): Gianfranco Spiteri, Johanna Takkinen, Andrew Amato.
