# Authors’ correction for Euro Surveill. 2018;23(33)

**DOI:** 10.2807/1560-7917.ES.2021.26.36.210909c

**Published:** 2021-09-09

**Authors:** Eurosurveillance editorial team

**Affiliations:** 1 European Centre for Disease Prevention and Control (ECDC), Stockholm, Sweden

In the article ‘Hepatitis A outbreak disproportionately affecting men who have sex with men (MSM) in the European Union and European Economic Area, June 2016 to May 2017’ by Ndumbi et al. published on 16 August 2018, a second affiliation, inadvertently omitted at the date of publication, was added for Ettore Severi. This correction was made on 8 September 2021 and the last affiliation was renumbered.

